# Objective approach to diagnosing attention deficit hyperactivity disorder by using pixel subtraction and machine learning classification of outpatient consultation videos

**DOI:** 10.1186/s11689-024-09588-z

**Published:** 2024-12-24

**Authors:** Yi-Hung Chiu, Ying-Han Lee, San-Yuan Wang, Chen-Sen Ouyang, Rong-Ching Wu, Rei-Cheng Yang, Lung-Chang Lin

**Affiliations:** 1 Department of Information Engineering, I-Shou University, No. 1, University Road, Yanchao District, Kaohsiung City, 824005 Taiwan; 2 Department of General Medicine, Shin Kong Wu Ho-Su Memorial Hospital, No. 95, Wenchang Road, Shilin District, Taipei City, 111045 Taiwan; 3 Department of Information Management, National Kaohsiung University of Science and Technology, No. 1, University Road, Yanchao District, Kaohsiung City, 824005 Taiwan; 4 Department of Electrical Engineering, I-Shou University, No. 1, Sec. 1, Syuecheng Road, Dashu District, Kaohsiung City, 84001 Taiwan; 5 Departments of Pediatrics, Kaohsiung Medical University Hospital, Kaohsiung Medical University, No. 100, Tzyou 1st Road, Sanmin District, Kaohsiung City, 80756 Taiwan; 6 Department of Pediatrics, School of Medicine, College of Medicine, Kaohsiung Medical University, No. 100, Shih-Chuan 1st Road, Sanmin District, Kaohsiung City, 807378 Taiwan

**Keywords:** Attention deficit hyperactivity disorder, Video analysis, Pixel subtraction, Machine learning, Swanson, Nolan, Pelham questionnaire

## Abstract

**Background:**

Attention deficit hyperactivity disorder (ADHD) is a common childhood neurodevelopmental disorder, affecting between 5% and 7% of school-age children. ADHD is typically characterized by persistent patterns of inattention or hyperactivity–impulsivity, and it is diagnosed on the basis of the criteria outlined in the *Diagnostic and Statistical Manual of Mental Disorders*,* Fifth Edition*, through subjective observations and information provided by parents and teachers. Diagnosing ADHD in children is challenging, despite several assessment tools, such as the Swanson, Nolan, and Pelham questionnaire, being widely available. Such scales provide only a subjective understanding of the disorder. In this study, we employed video pixel subtraction and machine learning classification to objectively categorize 85 participants (43 with a diagnosis of ADHD and 42 without) into an ADHD group or a non-ADHD group by quantifying their movements.

**Methods:**

We employed pixel subtraction movement quantization by analyzing movement features in videos of patients in outpatient consultation rooms. Pixel subtraction is a technique in which the number of pixels in one frame is subtracted from that in another frame to detect changes between the two frames. A difference between the pixel values indicates the presence of movement. In the current study, the patients’ subtracted image sequences were characterized using three movement feature values: mean, variance, and Shannon entropy value. A classification analysis based on six machine learning models was performed to compare the performance indices and the discriminatory power of various features.

**Results:**

The results revealed that compared with the non-ADHD group, the ADHD group had significantly larger values for all movement features. Notably, the Shannon entropy values were 2.38 ± 0.59 and 1.0 ± 0.38 in the ADHD and non-ADHD groups, respectively (*P* < 0.0001). The Random Forest machine learning classification model achieved the most favorable results, with an accuracy of 90.24%, sensitivity of 88.85%, specificity of 91.75%, and area under the curve of 93.87%.

**Conclusion:**

Our pixel subtraction and machine learning classification approach is an objective and practical method that can aid to clinical decisions regarding ADHD diagnosis.

## Background

Attention deficit hyperactivity disorder (ADHD) is a common childhood neurodevelopmental disorder characterized by an ongoing pattern of inattention or hyperactivity–impulsivity that leads to functioning or developmental problems [1]. Symptoms appear before age 12 and have potential to cause substantial impairments in multiple settings, such as school, work, and interactions with family or peers [2]. Such influences profoundly affect children’s academic achievement, well-being, and social interactions. ADHD is highly prevalent in children, adolescents, and young adults worldwide, affecting between 5% and 7% of children and adolescents, with a higher incidence in boys (boy: girl ratio in the range between 3:1 and 4:1) [3]. ADHD is not solely a childhood disorder; it often persists into adulthood and old age [2]. Because of the high incidence of adverse functional outcomes in ADHD, diagnosis and treatment of the disorder are critical clinical concerns [4]. The typical diagnostic procedures of ADHD employed by psychiatrists, neurologists, pediatricians, and family practitioners are primarily based on subjective assessments of perceived behavior. Since 1998, the American Medical Association has suggested that approaches to diagnosing ADHD involve (1) a comprehensive interview with the child’s adult caregivers, (2) a mental status examination of the child, (3) a medical examination for general health and neurological status, (4) an assessment of cognitive ability and achievement, (5) an ADHD-focused parent and teacher rating assessment conducted using specific scales, and (6) school reports and other adjunctive evaluations (e.g., speech or language assessment) [5]. Diagnosing ADHD in children remains challenging despite the widespread availability of various assessment tools, including the Swanson, Nolan, and Pelham (SNAP) questionnaire, the Vanderbilt ADHD Diagnostic Rating Scale, and the visual analog scale [6]. Of these, the SNAP questionnaire is the most frequently employed and comprises three subscales, that is, inattention, hyperactivity–impulsivity, and symptoms of oppositional defiant disorder subscales. These assessment tools are based on scale ratings assigned by teachers or parents and diagnoses made by specialists [1]. Hence, these scales only provide subjective perspectives and may lead to biased diagnoses. Until now, a scientific and objective tool for ADHD diagnosis needs to be established.

Image processing has long been employed in video surveillance [7]. Recently, image processing has been applied to investigate nuances in human movements [8]. Movement detection involves tracking moving objects by using an algorithm [7]. A common movement detection method is pixel subtraction of two consecutive gray images after separation of frames from a video. Devi et al. demonstrated that the frame subtraction method is an efficient alternative means of comparing image pixel values in subsequent frames captured 2 s apart when two frames are being used to detect movement. In this method, the first frame is used as a reference, and the second is used to calculate the movement of an object. The comparison of these two frames or images is performed by calculating differences in pixel values [8, 9]. Patients with ADHD often exhibit increased activity, and therefore, movement analysis may serve as an objective diagnostic tool for the condition [10, 11]. Therefore, the present study employed pixel subtraction and machine learning models to develop an objective diagnostic test for ADHD.

## Methods

### Participants

We included 43 children who had received a diagnosis of ADHD (24 boys and 19 girls, age [mean ± standard deviation (SD)]: 7 years 6 months ± 2 years 1 month) and 42 children who had not received a diagnosis of ADHD (21 boys and 21 girls, age [mean ± SD]: 7 years 9 months ± 2 years 2 months). No significant difference was observed between the ages of the children in the ADHD and non-ADHD groups. Diagnoses of ADHD were based on the *Diagnostic and Statistical Manual of Mental Disorders* (*DSM*)*-V* criteria, and ADHD severity was assessed using the SNAP IV. A continuous performance test (CPT) was used to measure the sustained and selective attention in patients with ADHD. Patients were excluded who had a history of severe intellectual disabilities, had abused drugs, had head injuries, or had received a diagnosis of psychotic disorders. The diagnoses in the patients without ADHD were headache, epilepsy, and dizziness, which were common in pediatric neurology. For each patient, a family member or legal guardian provided written informed consent for their child’s participation. The ethic regulations were conducted in accordance with the Declaration of Helsinki. Ethical approval was obtained from the Institutional Review Board of Kaohsiung Medical University Hospital [KMUIRB-SV (I)-20190060].

### Movement Recording and Analysis

**Fig. 1:**
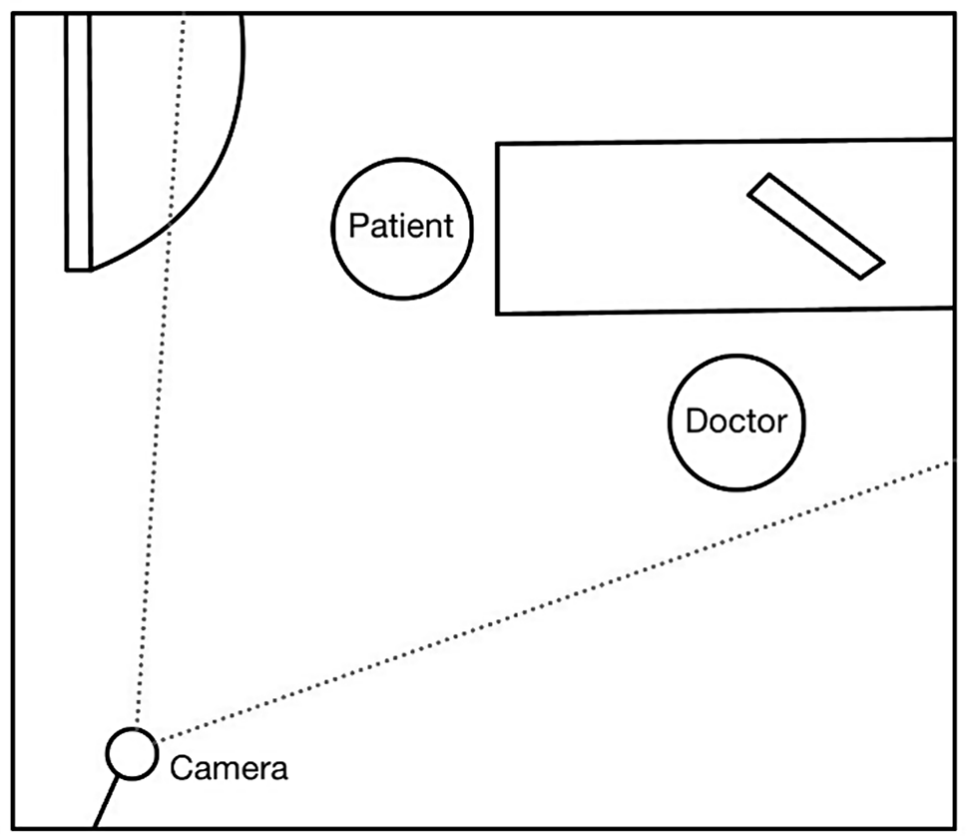
Video recorder view in the consultation room

**Fig. 2:**
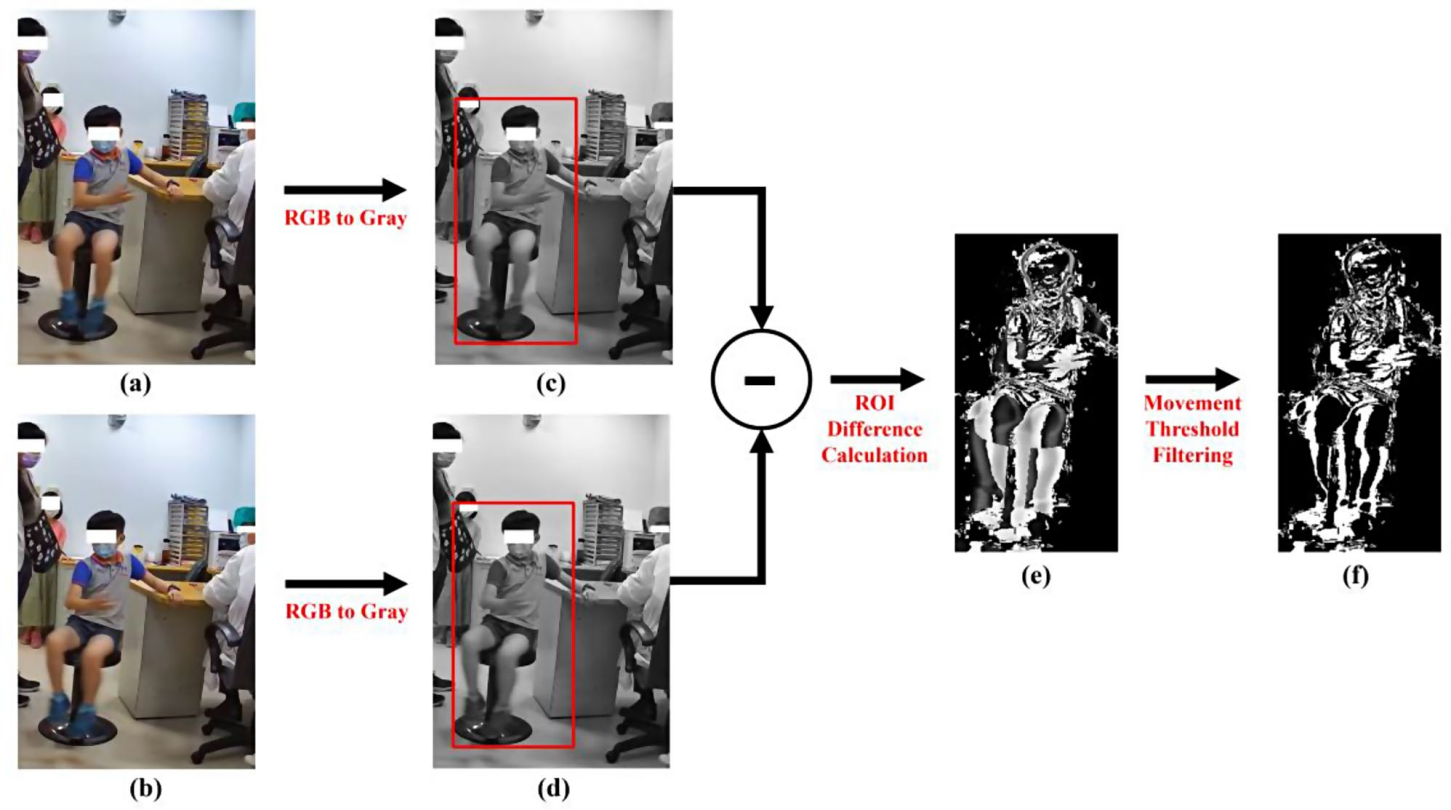
Diagram of the pixel subtraction method

We used pixel subtraction quantification to analyze video footage obtained during consultations with a pediatric neurologist. We used a two-dimensional camera (I-Family IF-005D) to record movement videos of each patient. The videos were captured at a sampling rate of 30 Hz and a resolution of 1280 × 720 pixels. The video recorder was placed in a fixed, unobtrusive position in the consultation room, as illustrated in Fig. 1. Our pixel subtraction method and movement analysis diagram are presented in Fig. 2. The input video frames, originally in color, were three-dimensional; we converted them to grayscale images. For example, consider the first two frames of the video sequence, referred to as the first frame ($\:{f}_{1}$) and the second frame ($\:{f}_{2}$). The original color images of these frames are shown in Fig. 2a and b, while their corresponding grayscale images are shown in Fig. 2c and d. This conversion significantly reduced computational time without compromising the results of the movement analysis. After obtaining a series of sequential grayscale images, pixel subtraction was performed pairwise for each consecutive image pair. Assuming the video was captured at a sampling rate of 30 Hz, the frames were numbered $\:({f}_{1},\:{f}_{2},\dots\:{f}_{30})\:$ for the first second of the video. Pixel subtraction for the first pair was calculated as $\:\left|{f}_{2}-{f}_{1}\right|$, followed by $\:\left|{f}_{3}-{f}_{2}\right|$, and so forth, up to $\:\left|{f}_{30}-{f}_{29}\right|$. This process was repeated for each consecutive frame pair that defines the temporal dimension of the video, resulting in a series of pixel-subtracted images tracked only within the region of interest (ROI), as shown in Fig. 2c and d. The ROI, depicted as the red rectangular region, was used to limit the analysis to the relevant area. The resulting pixel-subtracted image is shown in Fig. 2e. The final pixel-subtracted image was obtained by filtering with a significant movement threshold, shown in Fig. 2f. The detailed definitions of the ROI and the significance movement threshold will be explained in subsequent sections. The series of subtracted images that were obtained were used for movement analysis. Because each patient’s consultation time varied, the initial 4 min of video recording were employed for movement analysis to minimize comparison bias. When the patients visited the pediatric neurologist, they sat on a medical chair. If the child maintained a stable sitting posture over time, the pixel values for consecutive images did not markedly differ, and consequently, the calculated frame-by-frame subtraction values of the pixel subtraction were approximately zero. By contrast, if the patients exhibited fidgeting behavior, such as swaying or swiveling, the pixel values differed, resulting in large values in the calculated frame-by-frame subtraction. A previous study demonstrated that all measured human body movements are contained within the frequency of 20 Hz [12]. Therefore, to explore whether different sampling rates affect the performance of pixel subtraction and machine learning classification, we evaluated various sampling rates when implementing pixel subtraction. For example, let $\:Q=({f}_{1},{f}_{2},\dots\:,\:{f}_{30}$) be the sequence of frames of the first second in a video. If we obtain five subtracted images per second, the original 30 Hz video will be downsampled to 6 Hz. That is, after downsampling, $\:{Q}^{{\prime\:}}=\left({f}_{1}^{{\prime\:}},{f}_{2}^{{\prime\:}},\dots\:,{f}_{6}^{{\prime\:}}\right)=({f}_{1},{f}_{6},\dots\:,\:{f}_{30})$. The downsampling of the corresponding subtracted image sequence $\:{Q}^{{\prime\:}}$ was defined as follows.

1 $$\begin{aligned}{Q}^{{\prime\:}}&=({f}_{1}^{{\prime\:}},{f}_{2}^{{\prime\:}},\dots\:,{f}_{d-1}^{{\prime\:}})\\&=\left(\begin{aligned}&\left|{f}_{\frac{1\cdot\:S}{\left(d-1\right)\cdot\:F}}-{f}_{1}\right|,\left|{f}_{\frac{2\cdot\:S}{\left(d-1\right)\cdot\:F}}-{f}_{\frac{1\cdot\:S}{\left(d-1\right)\cdot\:F}}\right|,\cr&\quad\dots\:,\left|{f}_{\frac{(d-2)\cdot\:S}{\left(d-1\right)\cdot\:F}}-{f}_{\frac{(d-1)\cdot\:S}{\left(d-1\right)\cdot\:F}}\right|\end{aligned}\right)\end{aligned}$$

where $\:d$ represents the value of the downsampling rate; $\:S$ represents the value of the original sampling rate multiplied by the number of selected video frames, which is 30 Hz per second; and $\:F$ represents the value of the number of selected video seconds.

Accordingly, in downsampling to 6 Hz, five subtracted images are obtained per second, resulting in a 4-minute video comprising 1,200 consecutive subtracted images. In our approach, when no substantial movement difference was observed, the pixel values of any two consecutive images were approximately equal. Thus, the output pixel value was near zero after pixel subtraction, and the pixels in the output image were nearly opaque. By contrast, if any change or movement occurred between the capture of the two input images, the light portion of the subtracted image (Fig. 2e) indicated a movement difference. Using this pixel subtraction technique, we identified small movements in our participants that were imperceptible to the naked eye.

In the present study, movement was identified and tracked only within the ROI representing the participant’s movement in the subtracted images to avoid the influence of other individuals on the analytical results. Moreover, because each patient’s height varied, slight differences exist in the defined ROI for each patient. Therefore, we selected the corresponding ROI region from the subtracted image sequence $\:{Q}^{{\prime\:}}$ for each patient, obtaining the $\:ROI$ subtracted image sequence. The ROI is depicted as the red rectangular region as shown in Fig. 2c and d. We set a threshold $\:\theta\:$ for the pixel value. For example, if the difference in pixel values in the first subtracted image $\:\left|{Q}_{1}^{{\prime\:}}\left(i,j\right)\right|$ exceeded the threshold $\:\theta,$, $\:{ROI}_{1}\left(i,j\right)$ was set to 1; otherwise, it was set to 0. In dynamic image processing, all pixels in $\:{ROI}_{1}\left(i,j\right)$ with the value of 1 were considered to be the result of movement [13]. This process was then repeated for subsequent image sequences. The significance movement within the $\:{ROI}_{1}$, as shown in Fig. 2f, was defined as follows:

2 $$\:{ROI}_{k}\left(i,j\right)=\left\{\begin{array}{c}1,\:\:if\:{Q}_{k}^{{\prime\:}}\left(i,j\right)>\theta\:\\\:0,\:\:otherwise\end{array}\right.,\:\:\:\left(i,j\right)\in\:SA,\:\:\:k=\text{1,2},\dots\:,N$$

where $\:\:{ROI}_{k}\left(i,j\right)$ represents the pixel value in the $\:k$th frame within the ROI, with $\:i$ and $\:j$ representing the pixel $\:x$ and $\:y$ coordinates, respectively. $\:SA$ represents the set of image coordinates corresponding to the ROI, $\:N$ represents the number of subtracted images in the 4-minute video. $\:\theta\:$ represents the threshold value. Based on our experiment, the threshold pixel value $\:\theta\:$ is a constant value [13] and was set to 100, which was determined to represent significant movement.

The sum of the pixels in each subtracted frame was calculated to quantify the extent of patient movement in each subtracted frame. The resulting vector characterizes patient movement throughout the entire video. The sequence of movement along the measurement vector $\:M$ was defined as follows:

3 $$\:M=\left({m}_{1},{m}_{2},{m}_{3},\dots\:,{m}_{N}\right)$$

where

$$\:{m}_{k}={\sum\:}_{\left(i,j\right)\in\:SA}{ROI}_{k}\left(i,j\right),\:\:\:\:k=\text{1,2},\dots\:,N$$

where $\:{ROI}_{k}\left(i,j\right)$ represents the pixel value in the $\:k$th frame within the ROI, with $\:i$ and $\:j$ representing the pixel $\:x$ and $\:y$ coordinates, respectively.

Patients with ADHD often exhibit fidgeting behavior when seated or exhibit noticeable movement. This movement can be quantified through the mean ($\:\mu\:$), variance ($\:\stackrel{-}{var}$), and Shannon entropy ($\:\stackrel{-}{se}$), which were used in this study to analyze the movement vector.

Greater average movement indicated fidgeting. The mean movement in the sequence $\:M$ was defined as follows:

4 $$\:\mu\:=\frac{1}{N}{\sum\:}_{k=1}^{N}{m}_{k}$$

where $\:{m}_{k}$ represents the value of $\:M$ corresponding to the $\:k$th frame.

Greater variance in movement was considered to indicate greater fidgeting. To avoid the influence of outliers when calculating the variance directly from the movement sequence, we use a sliding window to calculate the variance for each time window, and then computes the average of the variances across all time windows. The averaged variance in movement of the sequence $\:M$ was defined as follows:

5 $$\:\stackrel{-}{var}=\frac{1}{L}{\sum\:}_{k=1}^{L}Var\left({M}_{k}\right),\quad\:L=N-W$$

where $$\:Var\left({M}_{k}\right)=\frac{1}{W}{\sum\:}_{i=1}^{W}{\left({m}_{j+i}-\stackrel{-}{{M}_{k}}\right)}^{2}$$

where $\:{M}_{k}=\left({m}_{j+1},{m}_{j+2},\dots\:,{m}_{j+W}\right)$, $\:j=\frac{W(k-1)}{2}$ represents the $\:k$th movement subsequence of $\:M$, with a window size of $\:W\:$and $\:L\:$indicates the number of movement subsequences. Additionally, the window and overlapping sizes were set to 5 and 2.5 s, respectively. $\:Var\left({M}_{k}\right)$ represents the variance of $\:{M}_{k}$, and $\:\stackrel{-}{{M}_{k}}$ represents the mean of $\:{M}_{k}$.

Greater entropy in movement was considered to indicate irregular and unpredictable patient movement. Accordingly, we used Shannon entropy to extract patient movement rhythm. Shannon entropy is used to calculate entropy on the basis of the probability distribution of movement. The higher the entropy value is, the greater the information content of the movement sequence and the greater the unpredictability and complexity of the movement are. To avoid the influence of outliers when calculating Shannon entropy directly from the movement sequence, we use a sliding window to calculate the Shannon entropy for each time window and then compute the average of the Shannon entropies across all time windows. The averaged Shannon entropy for movement of the sequence $\:M$ was defined as follows:

6 $$\:\stackrel{-}{se}=\frac{1}{L}{\sum\:}_{k=1}^{L}SE\left({M}_{k}\right),\quad\:L=N-W$$

where

$$\:SE\left({M}_{k}\right)=-{\sum\:}_{i=1}^{W}{P}_{{m}_{j+i}}{log}_{2}{(P}_{{m}_{j+i}})$$

where $\:SE\left({M}_{k}\right)$ represents the Shannon entropy of $\:{M}_{k},$ and $\:{P}_{{m}_{k+i}}$ represents the probability of occurrence of $\:{m}_{k+i}$ in the movement sequence $\:{M}_{k}$.

### Feature discriminability analysis

To evaluate the discriminability between the ADHD and non-ADHD groups in terms of each movement feature, we employed classification analyses based on six machine learning methods: support vector machines (SVM), random forest, decision tree, k-nearest neighbor (KNN), adaptive boosting (AdaBoost), and extreme gradient boosting (XGBoost). The machine learning library scikit-learn was utilized for comparative analysis [14]. We employed nested cross-validation to optimize the model hyperparameter and evaluate the model’s classification performance. The outer loop employs 10-fold cross-validation for model training and testing. During each iteration of the outer loop, 1-fold of ADHD and non-ADHD patients’ movement features are used as the test dataset, while the remaining 9-fold of ADHD and non-ADHD patients’ movement features are used as the training dataset. The training dataset obtained from each outer loop iteration is then used for model hyperparameter optimization and model training. The hyperparameter optimization uses grid search and 5-fold cross-validation to identify the suitable parameters for each machine learning classification model. Therefore, the grid search for tuning the hyperparameters of each classification model was defined as follows:

1. Support vector machine (SVM): The kernel type of the SVM was specified as the radial basis function. The model parameters “gamma” and “C” were optimized using a grid search within the ranges {50, 100, 300, 500} and {0.001, 0.01, 0.1, 1}, respectively. Default values were used for all other parameters in the library.
2. Random forest: The model parameter “n-estimators” was optimized using a grid search within the ranges {1, 5, 10, 20, 30, 50}. Default values were used for all other parameters in the library.
3. Decision tree: The tree algorithm of the decision tree was specified as the CART. The model parameter “max-depth” was optimized using a grid search within the ranges {1, 2}. Default values were used for all other parameters in the library.
4. K-nearest neighbor (KNN): The model parameter “n-neighbors” was optimized using a grid search within the ranges {1, 2}. Default values were used for all other parameters in the library.
5. Adaptive boosting (AdaBoost): The weak classifiers of the AdaBoost was specified as the CART tree algorithm. The model parameter “n-estimators” was optimized using a grid search within the ranges {1, 5, 10, 20, 30, 50}. Default values were used for all other parameters in the library.
6. Extreme gradient boosting (XGBoost): The weak classifiers of the AdaBoost was specified as the CART tree algorithm. The model parameters “max-depth”, “learning rate” and “n-estimators” were optimized using a grid search within the ranges {1, 2}, {0.1, 0.2}, and {1, 5, 10, 20}, respectively. Default values were used for all other parameters in the library.

To minimize biased comparisons of classification results among different movement features with different machine learning classification models, the resampling strategy of nested cross-validation was repeated 10 times. Consequently, a total of 100 pairs of training and testing datasets were obtained. The training datasets were used to train each classification model, and the testing datasets were used to evaluate the classification performance of the trained classification model.

### Feature error estimation and analysis

To evaluate the reliability of classification performance metrics for different movement features across various machine learning classification models, we calculated the standard error of the mean (SEM) [15] for each classification model and movement feature. Although nested cross-validation was repeated 10 times in this study, to mitigate potential overfitting and identify the movement feature with the best performance and the smallest error range across sampling iterations, we further quantified this variability. Therefore, SEM was employed to quantify the variability in classification performance across multiple iterations of cross-validation, providing error estimates for the different performance metrics of each classification model. The SEM for classification performance metrics across multiple iterations of cross-validation was defined as follows:

7 $$\:SEM=\frac{\sigma\:}{\sqrt{n}}$$

where $\:\sigma\:$ represents the standard deviation of one of the performance metrics across multiple iterations of cross-validation, and $\:n$ represents the number of iterations, which was set to 100 in this study, corresponding to the total number of training and testing dataset pairs.

### Statistical analysis

All statistical analyses were conducted using SAS v. 9.4; (SAS Institute, Cary, NC, USA). The results are presented as means ± SDs. Patients with ADHD and non-ADHD were compared in terms of their movement features by using the two-sample *t-test* and their sex distribution was compared by using chi-squared test. A *P* value < 0.05 was considered significant.

## Results

No significant difference in patient sex and age were observed between the ADHD and non-ADHD groups. Table 1 presents the average score (mean ± SD) on the SNAP IV rating scale assessed by parents and teachers. No SNAP rating was available for the non-ADHD group. In total, 20 boys had ADHD-C, 2 boys had ADHD-I, and 2 boys had ADHD-H; 14 girls had ADHD-C, and 5 girls had ADHD-I. According to the literature, ADHD-C and ADHD-H are the most prevalent subtypes of ADHD (78.0–81.7%), followed by ADHD-I (18.3–22.0%) [16–18]. In the present study, 36 of the 43 patients had ADHD-C or ADHD-H. Therefore, most of the recruited patients exhibited combined or hyperactivity symptoms. The SNAP IV total scores obtained from parents and teachers were 34.16 ± 18.37 and 31.09 ± 18.31, respectively. To compare the classification results between the ADHD and non-ADHD groups that were obtained using various downsampling rates, we conducted experiments in which we downsampled the original video from 30 Hz to 15 Hz or 6 Hz or retained the original sampling rate of 30 Hz. We subsequently used a *t* test to compare three movement features between the ADHD and non-ADHD groups. The results are presented in Table 2. The ADHD group had higher values for all three movement features, and the movement features significantly differed between the ADHD and non-ADHD groups. Notably, the Shannon entropy values were 2.38 ± 0.59 and 1.0 ± 0.38 in the ADHD and non-ADHD groups, respectively (*P* < 0.0001). The machine learning classification model achieved the most favorable results, with an accuracy of 90.24%, sensitivity of 88.85%, specificity of 91.75%, and area under the curve of 93.87%. Additionally, we confirmed the results of the statistical comparison by utilizing visual observations to determine whether patients were seated calmly or exhibiting noticeable movement. We also compared the sensitivity of SNAP scores, CPT, and our proposed method in patients with ADHD. The sensitivities of SNAP of inattention and hyperactivity scores from parents were 54.84% and 51.61%. The sensitivities of SNAP of inattention and hyperactivity scores from teachers were 38.71% and 41.94%. The sensitivities of CPT parameters were all less than 50.00%. Compared with SNAP and CPT, our proposed method had better performance in terms of sensitivity with 92.00%.

**Table 1:** Demographic data of patients with ADHD and Non-ADHD

	ADHD	Non-ADHD	*p* Value
Sex (M/F)^⊚^	24/19	21/21	0.749
Age*	7y6m ± 2y1m	7y9m ± 2y2m	0.476
***Parent’s SNAP score***			
Inattention Hyperactivity Oppositional Total	13.69 ± 5.31 13.46 ± 6.92 10.51 ± 6.72 34.16 ± 18.37	N/A	N/A
***Teacher’s SNAP score***			
Inattention Hyperactivity Oppositional Total	15.17 ± 6.67 11.74 ± 7.39 7.35 ± 6.59 31.09 ± 18.31	N/A	N/A

*Note* Statistical analyses were performed using the ^⊚^chi-squared test, * two-sample t-test

**Table 2:** Statistical comparison of three movement features between ADHD and non-ADHD groups was conducted by different sampling rates

Movement feature (units)	Sampling rate (Hz)	ADHD	Non-ADHD	*p* Value
Mean (pixel)	6	1596.2 ± 1076.1	377.5 ± 196.1	< 0.0001
	15	625.5 ± 497.1	119.9 ± 65.3	< 0.0001
	30	309.2 ± 205.6	55.9 ± 28.5	< 0.0001
Variance (pixel^2^)	6	35.9 × 10^5^ ± 46.9 × 10^5^	7.8 × 10^5^ ± 8.4 × 10^5^	0.0002
	15	17.6 × 10^5^ ± 22.5 × 10^5^	4.7 × 10^5^ ± 8.4 × 10^5^	0.0014
	30	11.5 × 10^5^ ± 15.5 × 10^5^	4.0 × 10^5^ ± 7.1 × 10^5^	0.0065
Shannon entropy (bits)	6	4.1 ± 0.5	2.5 ± 0.7	< 0.0001
	15	3.6 ± 0.8	1.6 ± 0.6	< 0.0001
	30	2.3 ± 0.5	1.0 ± 0.3	< 0.0001

**Fig. 3:**
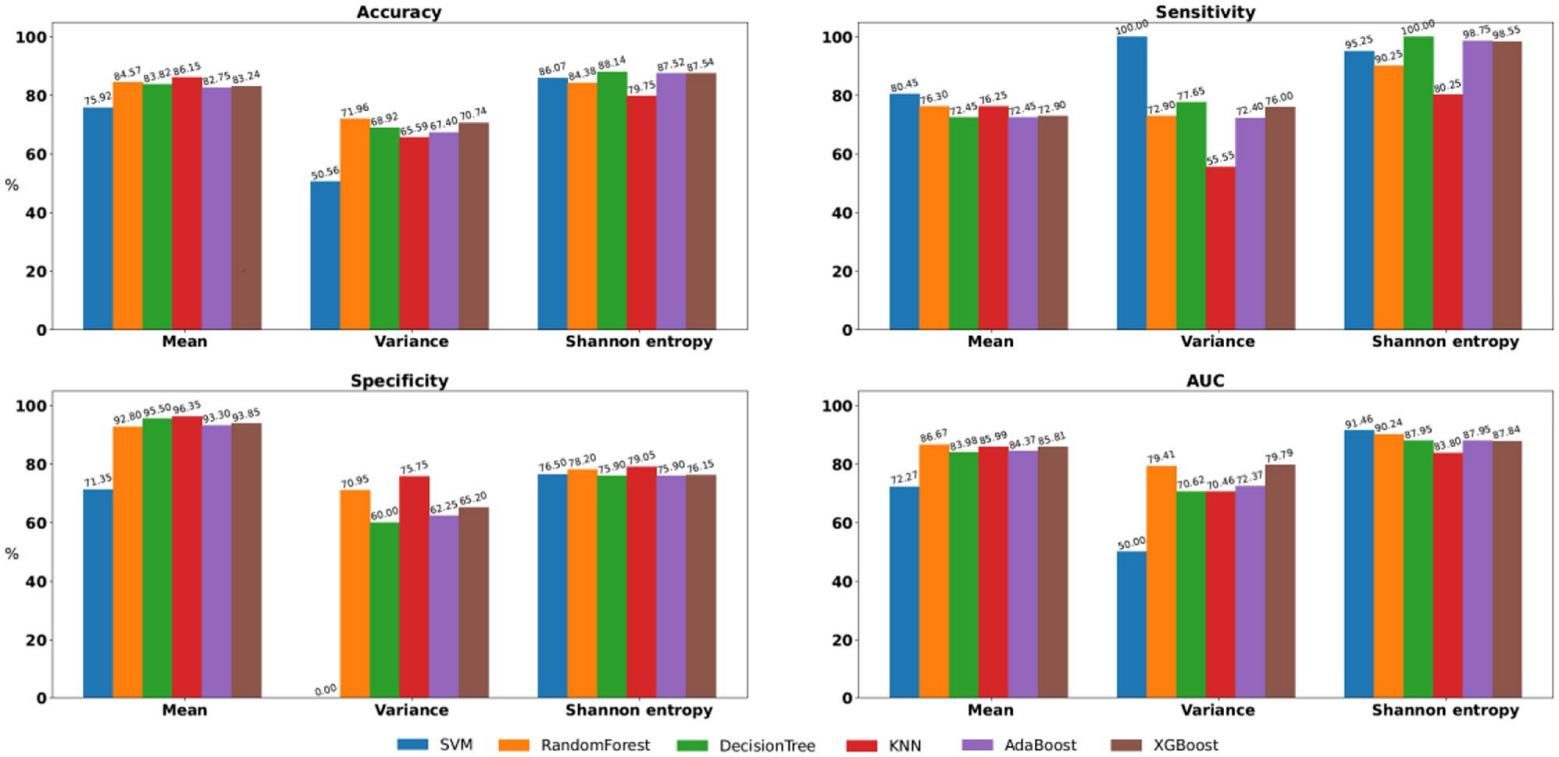
Comparisons of classification test performance metrics, specifically, of accuracy, sensitivity, specificity, and area under the curve, were conducted by downsampling the images to 6 Hz across classification models in all feature sets

**Fig. 4:**
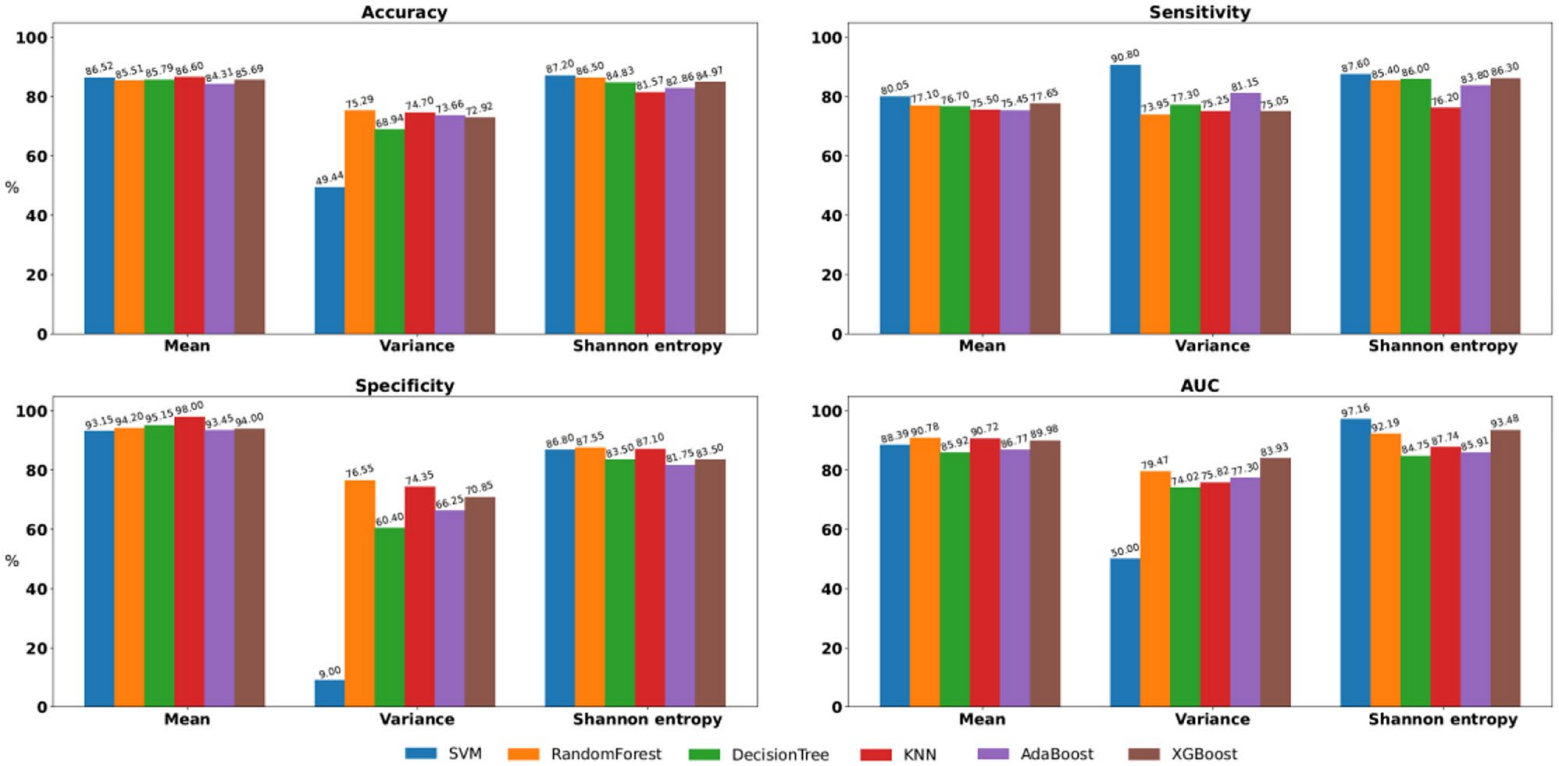
Comparisons of classification test performance metrics, specifically, of accuracy, sensitivity, specificity, and area under the curve, were conducted by downsampling the images to 15 Hz across classification models in all feature sets

**Fig. 5:**
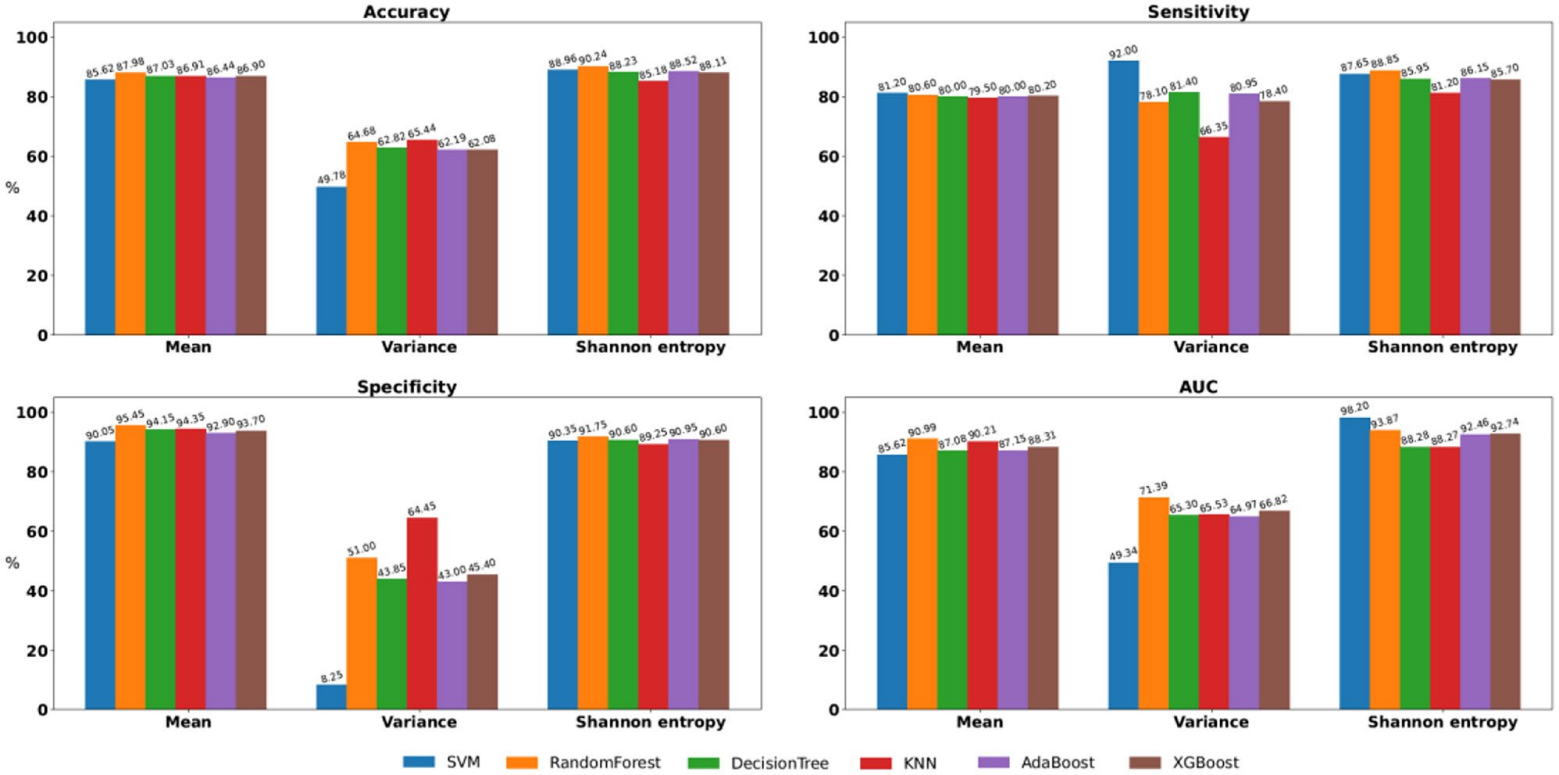
Comparisons of classification test performance metrics, specifically, of accuracy, sensitivity, specificity, and area under the curve, were conducted using the original image sampling rate of 30 Hz across classification models in all feature sets

Figures 3 and 4, and 5 present comparisons of the four classification test performance metrics, that is, accuracy, sensitivity, specificity, and area under the curve (AUC), obtained using six classification models for each movement feature. For downsampling to 6 Hz, “Decision Tree + Shannon entropy” exhibited the highest accuracy (88.14%), “SVM + Variance” and “Decision Tree + Shannon entropy” exhibited the highest sensitivity (100.0%), “KNN + Mean” exhibited the highest specificity (96.35%), and “SVM + Shannon entropy” exhibited the highest AUC (91.46%). For downsampling to 15 Hz, “SVM + Shannon entropy” exhibited the highest accuracy (87.20%), “SVM + Variance” exhibited the highest sensitivity (90.80%), “KNN + Mean” exhibited the highest specificity (98.00%), and “SVM + Shannon entropy” exhibited the highest AUC (97.16%). In retaining the original sampling rate of 30 Hz, “Random Forest + Shannon entropy” exhibited the highest accuracy (90.24%), “SVM + Variance” exhibited the highest sensitivity (92.00%), “Random Forest + Mean” exhibited the highest specificity (95.45%), and “SVM + Shannon entropy” exhibited the highest AUC (98.20%).

To identify the sampling rate with the optimal classification metrics for comparing individuals with ADHD and those without, we compared the discriminative ability of each sampling rate between the ADHD and non-ADHD groups. Of the three sampling rates and four performance indices, the 6 Hz sampling rate ranked first in sensitivity, the 15 Hz sampling rate ranked first in specificity, and the 30 Hz sampling rate ranked first in accuracy and AUC. The original sampling rate of 30 Hz achieved the most favorable results, that is, it ranked first for two performance indices, and its sensitivity and specificity were close to the best results obtained at 6 Hz and 15 Hz. To identify which movement features could distinguish between individuals with ADHD and those without at the original sampling rate of 30 Hz, we averaged the rankings of all movement features corresponding to each classification performance index. The optimal features are presented in Table 3. Shannon entropy ranked first in accuracy, sensitivity, and AUC, and the mean ranked first in specificity. Thus, Shannon entropy was the single feature that best discriminated between those with ADHD and those without. To determine the optimal Shannon entropy and original sampling rate of 30 Hz for classifying the ADHD and non-ADHD groups, we ranked Shannon entropy for each classification performance index across six machine learning models. Of the six machine learning models and four performance indices, the random forest ranked first in accuracy, sensitivity, and specificity, and the SVM ranked first in AUC. The ranking results are presented in Table 4. The Random Forest classification model achieved the most favorable results, with an accuracy, sensitivity, specificity, and AUC of 90.24%, 88.85%, 91.75%, and 93.87%, respectively.

**Table 3:** Averaged ranking of all movement features corresponding to each classification performance index by an original sampling rate of 30 hz

Features	Accuracy average rank	Sensitivity average rank	Specificity average rank	AUC average rank
Mean	1.8	2.5	**1.1**	1.8
Variance	3.0	2.3	3.0	3.0
Shannon entropy	**1.1**	**1.1**	1.8	**1.1**

**Table 4:** The ranking of Shannon Entropy for each classification performance index between six machine learning models by an original sampling rate of 30 hz

Classification models	Accuracy	Sensitivity	Specificity	AUC
SVM	2	2	5	**1**
Random forest	**1**	**1**	**1**	2
Decision tree	4	4	3	5
KNN	6	6	6	6
AdaBoost	3	3	2	4
XGBoost	5	5	3	3

**Fig. 6:**
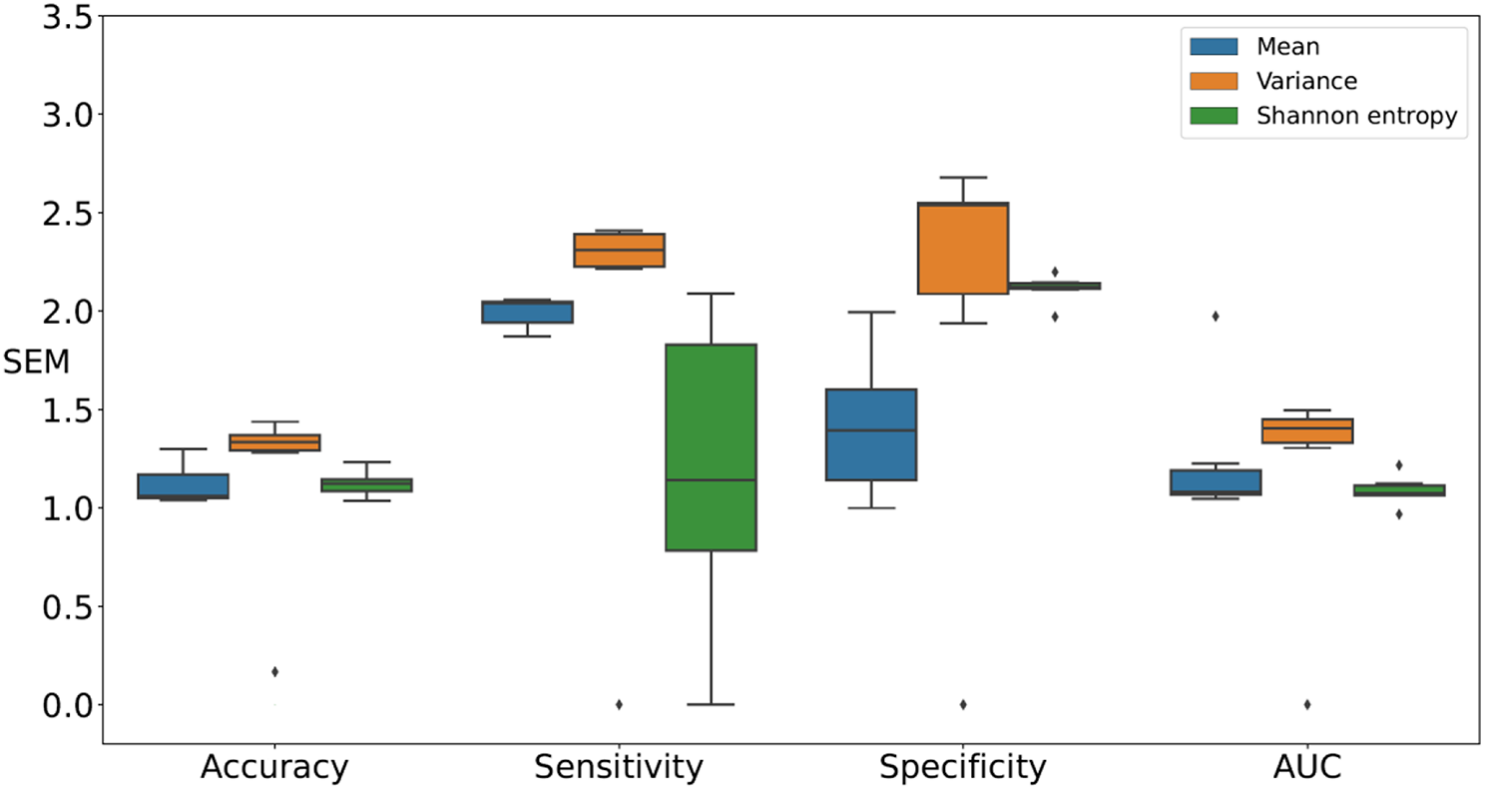
Comparisons of the SEM for performance metrics, specifically accuracy, sensitivity, specificity, and AUC, were conducted by downsampling the images to 6 Hz across classification models in all feature sets

**Fig. 7:**
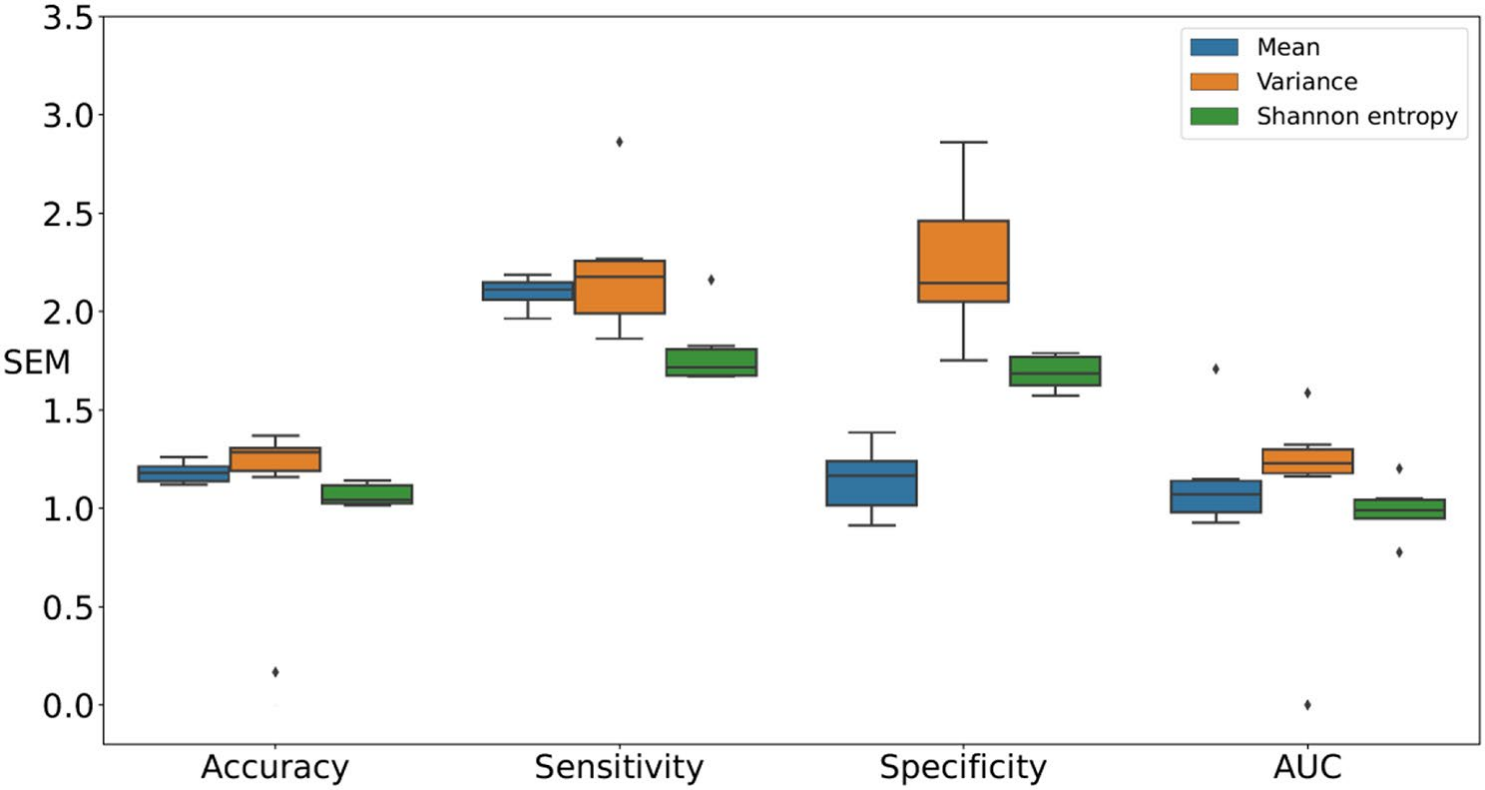
Comparisons of the SEM for performance metrics, specifically accuracy, sensitivity, specificity, and AUC, were conducted by downsampling the images to 15 Hz across classification models in all feature sets

**Fig. 8:**
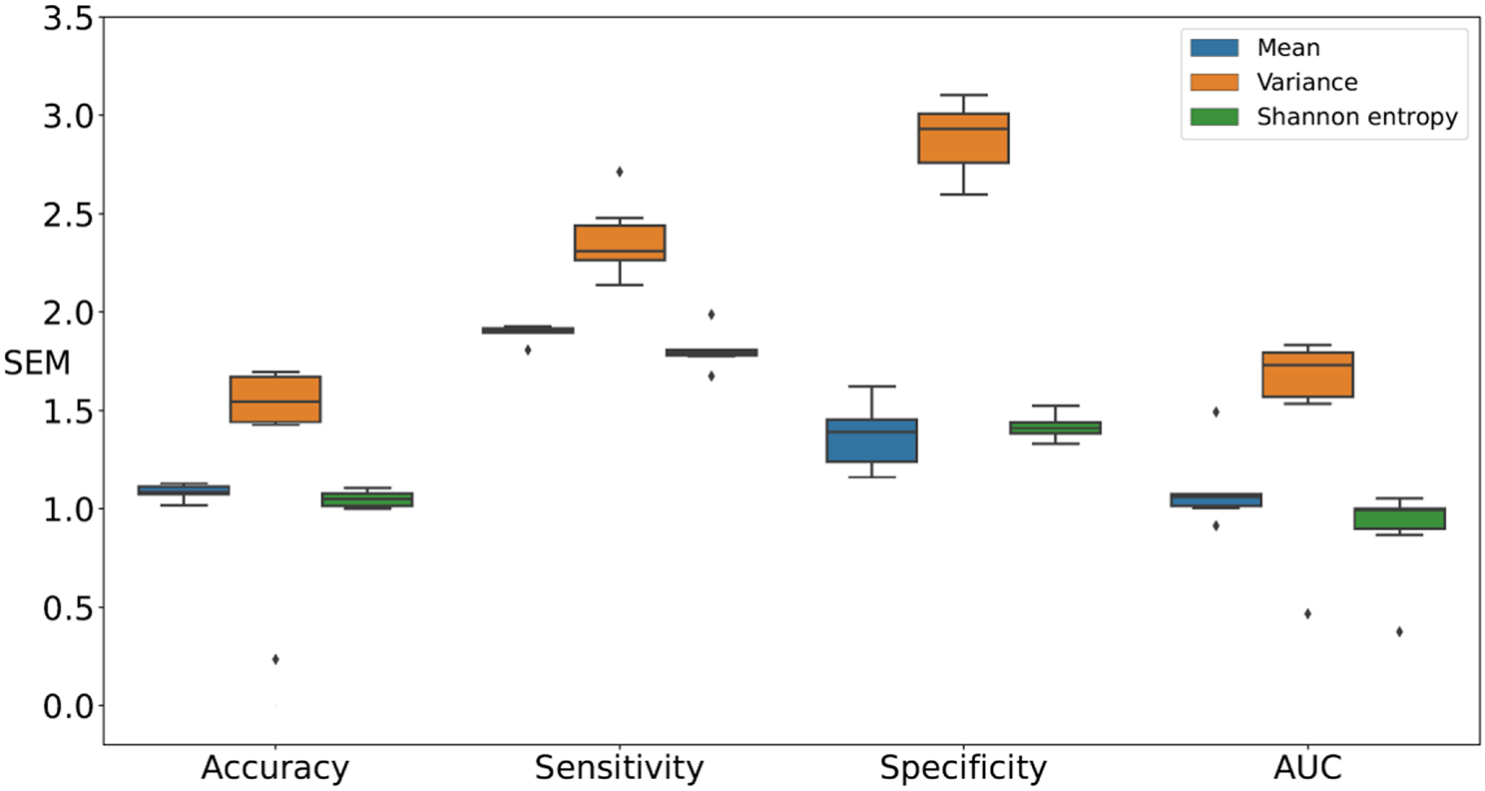
Comparisons of the SEM for performance metrics, specifically accuracy, sensitivity, specificity, and AUC, were conducted using the original image sampling rate of 30 Hz across classification models in all feature sets

Figures 6 and 7, and 8 present comparisons of the SEM for four classification test performance metrics, that is, accuracy, sensitivity, specificity, and AUC, obtained using six classification models for each movement feature. Note that each boxplot illustrates the SEM for each classifier across different movement features. For downsampling to 6 Hz, Shannon entropy exhibited lower variability compared to the Mean in accuracy and AUC. For sensitivity, Shannon entropy exhibited lowest SEM but with noticeably higher variability. For specificity, Mean achieved the lowest SEM. In contrast, Variance exhibited larger error margins and greater variability in specificity, as shown in Fig. 6. For downsampling to 15 Hz, Shannon entropy exhibited relatively low SEM and variability compared to the Mean in accuracy, sensitivity, and AUC. For specificity, Mean exhibited the lowest SEM. In contrast, Variance exhibited larger error margins and greater variability in sensitivity and specificity, as shown in Fig. 7. In retaining the original sampling rate of 30 Hz, Shannon entropy exhibited relatively low SEM for accuracy, sensitivity, and AUC. For specificity, Shannon entropy exhibited the lowest SEM variability compared to Mean. In contrast, Variance exhibited larger error margins and greater variability in four performance metrics, as shown in Fig. 8. Thus, Shannon entropy demonstrated the most consistent performance across different sampling rates, achieving relatively low SEM and variability in accuracy, sensitivity, and AUC. Additionally, it exhibited the lowest SEM variability in specificity at higher sampling rates, further supporting its reliability for distinguishing between individuals with ADHD and those without.

## Discussion

This study revealed that using the pixel subtraction method and Shannon entropy to quantify the movement of individuals with and without ADHD can provide an objective ADHD diagnosis. The Shannon entropy value was substantially higher in the ADHD group than in the non-ADHD group. The experimental results indicate that retaining the original sampling rate of 30 Hz, “Random Forest + Shannon entropy” yielded the optimal results, with an accuracy, sensitivity, specificity, and AUC of 90.24%, 88.85%, 91.75%, and 93.87%, respectively. The main reason for this excellence was the highly discriminative nature of Shannon entropy extracted from the patient movement videos between the ADHD and non-ADHD groups, resulting in well-trained classification models and corresponding superior prediction capability. Further analysis demonstrated that Shannon entropy achieved the most consistent performance across all three sampling rates, with relatively low SEM and variability in accuracy, sensitivity, and AUC. Moreover, it exhibited the lowest SEM variability in specificity at higher sampling rates, further supporting its reliability for distinguishing between the ADHD and non-ADHD groups. Therefore, Shannon entropy is a useful and objective marker for diagnosing ADHD. Additionally, the movement features significantly differed between the ADHD and non-ADHD groups at all three sampling rates. The machine learning classification results demonstrate that increasing the sampling rate yielded superior discriminatory power. According to the Nyquist–Shannon sampling theorem [19], if body movements are contained within a frequency of 20 Hz [12], a sampling rate of at least 40 Hz is required to capture movement changes. Therefore, our classification results are consistent with those of a previous study on human body movements [12]. In other words, we require a higher sampling rate to capture more subtle or rapid movement changes. Although a lower sampling rate can reduce the cost and time required for analysis, it may result in an inability to capture some rapid movements.

Because the chair in the consultation room could be rotated by patients, body spinning and lower extremity movements were the predominant movements. Schworm et al. demonstrated that children with ADHD often trail their hands across tables, reaching for objects aimlessly [20]. The ADHD group also exhibited a higher rate of leg and foot movements, such as swinging, tapping, or shaking, than did the non-ADHD group. Children with ADHD often turn their heads when they shift their attentional focus to a different stimulus [21].The experimental results demonstrate that Shannon entropy provided the best classification result between the ADHD and non-ADHD groups. According to previous studies, the movements of children with ADHD include upper and lower extremity movements [20, 21]. In the current study, the other features (mean and variance), which describe average movement and pronounced movement, were less helpful in distinguishing between children with ADHD and those without. This indicates that children with ADHD exhibit more irregular and unpredictable movement behavior in the consulting room.

The clinical diagnosis of ADHD depends heavily on the professional expertise of doctors and is primarily assessed using observation and information provided by parents and teachers. This highly subjective diagnostic evaluation procedure often leads to inconsistent results. In addition, the *DSM-V* criteria used by diagnosing physicians to determine whether the child is more or less prone to a high degree of movement are subjective. Developing an objective method for capturing, identifying, and analyzing the movement patterns of children with ADHD is crucial to reducing the influence of subjectivity in such diagnoses. A previous study attempted to develop objective diagnostic measures to minimize the incidence of misdiagnosis of ADHD [22]. Based on the results recording the amount of movement in both ADHD and non-ADHD children, we detected some significant features. Comparing these two groups (ADHD versus non-ADHD), the results revealed significant differences in movement analysis, with the average movement higher in the ADHD group. This suggests that movement is one of the important features in distinguishing ADHD children from non-ADHD children. By review of most studies, children with ADHD exhibited more physical movement than those without ADHD. Our findings of increased movements in children with ADHD are consistent with those of that study [22]. Hyperactive children typically exhibit increased physical movements and an inability to sit still, especially in calm or quiet environments [23]. These behaviors are core symptoms of hyperactivity and impulsivity. For over a decade, studies have attempted to record patient movements objectively. Most studies have employed motion capture by using accelerometers or infrared devices placed on the body. One study used a Kinect camera to analyze the movement of patients and identified significant differences in the amount of objective movement between a group of children with ADHD and a control group. The ADHD group in that study had higher average movement values for all analyzed joints [24]. Another study used video analysis to verify that children with ADHD move more than children without ADHD when seated [25]. As demonstrated in the present study, objective measures with high specificity and sensitivity can improve accuracy in identifying quantifiable target symptoms. These findings also demonstrate that comparing video images of children who may have ADHD can assist clinicians in addressing the challenge posed by the often substantial differences between teacher and parent ratings in terms of SNAP IV scores for children’s behavior.

Several movement detection tools are available to assist physicians in diagnosing ADHD, including accelerometers, actigraphy, infrared recording, and ultra-wideband radar. Accelerometers and actigraphy are worn on the wrist or ankle and can be used at home or school instead of in a laboratory [26]. However, they do not function unless attached to the patient’s body, which limits their ecological validity. Additionally, only the movement of body parts to which sensors have been attached can be recorded. Furthermore, accelerometers analyze patient movements during normal daily activities and have limited battery life [27], whereas actigraphy are used to study patient sleep efficiency and are limited by a low sampling rate [28]. Regarding to infrared, the strength of infrared is noncontact without placing any type of sensor in the body of the subjects [29]. However, infrared detection is easily disrupted by light or other noise. Additionally, infrared recording requires the use of special detection and software equipment. Ultra-wideband radar is another noncontact method for recording movements that can be used in various situations, such as during a test or in a naturalistic setting [30]. The disadvantages of ultra-wideband radar are that it must be used in a restricted space and can be disrupted by surrounding objects. By contrast, the method employed in the present study is noncontact and uses video from a regular camera for analysis and the computation complexity is low. This method can accurately distinguish between individuals with and without ADHD following a short observation period. Additionally, detection can be conducted during regular consultations and does not affect normal behavior. Nevertheless, the weaknesses of our method are twofold: (1) the detection data may be occluded by other human bodies, and (2) the method must be used in a restricted space. However, in our consultation room, these two shortcomings were overcome through experimental design. The motion data we analyzed were limited to the red rectangular region defined in Fig. 2. The design will minimize the detection data interfered by other human bodies during consultations. It will reduce unwanted data to be included in our analysis. In addition, we used Shannon entropy as a classification feature between the ADHD and non-ADHD groups. This feature could depict the motion characteristics in patients with ADHD, such as irregular and unpredictable movement behavior in the consulting room. Furthermore, an appropriate machine learning model is also important for diagnostic discriminability. In the present study, the random forest classification model achieved the most favorable results. The aforementioned factors could explain that our method has better performance compared to existing efforts to achieve diagnostic discriminability based on motion capture.

Our study has some limitations. First, the cohort primarily comprised patients with ADHD-C; thus, our results may not be representative of all ADHD subtypes. Second, the movements of patients with ADHD in the consultation room may be affected by nonpharmacological factors, such as food intake on the day of assessment, sleep quality before assessment, and familiarity with the consultation environment. Future studies should include a questionnaire to investigate the relationship between these confounding factors and children’s movements. Third, the camera position and angle are critical components in movement analysis. Varying camera angles or positions can affect image quality or even lead to failure to detect movement, resulting in inaccuracy or bias in movement analysis. Future studies should use two or more cameras mounted at different locations in the room to address this limitation.

## Conclusions

The present study primarily included patients with ADHD who had received a diagnosis of the ADHD-C or ADHD-H subtypes. Our pixel subtraction movement quantization analysis of Shannon entropy in outpatient consultation room videos effectively distinguishes between children with ADHD and those without. Our results also reveal that compared with the non-ADHD group, the ADHD group exhibited substantially larger values for all movement features. Shannon entropy was particularly effective in distinguishing between the movements of patients in the ADHD and non-ADHD groups. In conclusion, the proposed machine learning approach is a reliable model for objectively determining whether a patient is likely to have ADHD. Because most patients with ADHD have either hyperactive or combined subtypes and exhibit symptoms of hyperactivity, our approach can aid physicians in making clinical decisions regarding ADHD diagnosis.

## Data Availability

No datasets were generated or analysed during the current study.

## Abbreviations

ADHD: Attention deficit hyperactivity disorder
SD: Standard deviation
SNAP: Swanson, Nolan, and Pelham Rating Scale
DSM: Diagnostic and Statistical Manual of Mental Disorders
ROI: Region of interest
SVM: Support vector machine
KNN: k-nearest neighbors
AdaBoost: Adaptive boosting
XGBoost: Extreme gradient boosting
CART: Classification and regression tree
AUC: Receiver operating characteristic curve
